# Links between Hobbies, Activities, Volunteering, and Cognitive Function in Mexican Older Men and Women

**DOI:** 10.1093/geroni/igaf122.509

**Published:** 2025-12-31

**Authors:** Joseph Saenz, Yuan Zhang, Erika Beidelman, Yang (Claire) Yang

**Affiliations:** Arizona State University, Phoenix, Arizona, United States; Columbia University, New York, New York, United States; Columbia University Mailman School of Public Health, New York, New York, United States; The University of North Carolina at Chapel Hill, Chapel Hill, North Carolina, United States

## Abstract

Engagement in cognitively stimulating activities throughout life may support cognitive function and help prevent decline. Social participation is linked to better cognitive outcomes in older adults in high-income countries; however, benefits may be more pronounced in populations who had historically limited access to stimulating opportunities in early-life (e.g., wide access to high-quality education). We used data from the 2015 Mexican Health and Aging Study and 2016 Mex-Cog study, including comprehensive neuropsychological tests assessing multiple cognitive domains (n = 1,968). We considered both the number of activities in two activity domains: hobbies and indoor activities, and volunteering and community activities, and individual activities within each domain. We employed linear regression models to assess relationships across cognitive domains. Hobbies and indoor activities were associated with better memory, language, visuospatial ability, executive function, and orientation. Volunteering and community activities showed no significant associations. Engagement in all individual hobbies and indoor activities, except watching television, were linked to better cognitive functions. Education-stratified analyses revealed that activities such as talking to friends, performing home maintenance, and attending sporting clubs were associated with better cognitive function among individuals with lower education but not among those with higher education. These associations were largely consistent between men and women. Findings provide nuanced evidence on the role of leisure and social activities in cognitive function among older Mexican adults, highlighting similarities and differences across education. Moving forward, we will examine health-related selection into activity participation using prior waves and use 2021 Mex-Cog data to assess changes across domains over five years.

